# NFC Internal: An Indoor Navigation System

**DOI:** 10.3390/s150407571

**Published:** 2015-03-27

**Authors:** Busra Ozdenizci, Vedat Coskun, Kerem Ok

**Affiliations:** NFC Lab-Istanbul, Department of Information Technologies, ISIK University, Istanbul 34980, Turkey; E-Mails: busraozdenizci@isikun.edu.tr (B.O.); keremok@isikun.edu.tr (K.O.)

**Keywords:** Near Field Communication, indoor navigation, indoor positioning, NFC Internal, mobile

## Abstract

Indoor navigation systems have recently become a popular research field due to the lack of GPS signals indoors. Several indoors navigation systems have already been proposed in order to eliminate deficiencies; however each of them has several technical and usability limitations. In this study, we propose NFC Internal, a Near Field Communication (NFC)-based indoor navigation system, which enables users to navigate through a building or a complex by enabling a simple location update, simply by touching NFC tags those are spread around and orient users to the destination. In this paper, we initially present the system requirements, give the design details and study the viability of NFC Internal with a prototype application and a case study. Moreover, we evaluate the performance of the system and compare it with existing indoor navigation systems. It is seen that NFC Internal has considerable advantages and significant contributions to existing indoor navigation systems in terms of security and privacy, cost, performance, robustness, complexity, user preference and commercial availability.

## 1. Introduction

Navigation is the process of monitoring and controlling the movement of an item from an origin to a destination along a path. Navigation systems provide reading, controlling and updating the movement of one’s position and guiding by intelligible visual, audible or tangible means while she is travelling on an intended route. If any diversion to outside of the route occurs, reorientation or correction to the destination is required [1]. Knowledge of position, called as positioning, is a must for accurate navigation, since it may be reached by extensive effort after repetitive useless corrective travels or else missing the target as the worst case. Human knowledge, or intuitive, might be helpful in navigation efforts; but technical contribution is obviously very much helpful.

Navigation systems are mostly based on Global Positioning System (GPS), which provides reliable location information on the Earth in almost all weather conditions and at all times with the help of multiple satellites [2,3]. GPS satellites are dedicated to positioning, and they orbit around the world solely for this purpose. Making use of GPS satellites for positioning has some requirements though. The foremost condition is that the navigating device has to receive signals from a specific number of satellites—four of them for the best result—to get the position with high accuracy. The signal level between the device and the satellite is normally not hindered by bad weather conditions, but line of sight between the device and the satellite is still a must. This implicitly puts forward the requirement for the device to be outdoors, *i.e.*, out of any buildings. Hence, use of the GPS system is called outdoor navigation.

Contrary to the wide usage of GPS in outdoor navigation, GPS receivers cannot perform satisfactorily in indoor environments because of the absence of line of sight to the satellites [4]. Additionally, indoor navigation in general, is much more complex when compared with well-established outdoor alternatives. As mentioned by [5], indoor environments such as buildings, university campuses and hospitals include various obstacles (*i.e.*, building geometry, walls, equipment), which reduce the propagation capability of electromagnetic waves; hence interference and noise originating from wired and wireless networks within the structures degrade the accuracy of positioning as well. Thus more accurate and reliable indoor navigation systems that will not be affected by the discouraging properties of indoor environments are needed in the next generation of navigation technologies.

Indoor navigation systems are designed to navigate the user within closed environments such as hospitals, gymnasiums, or schools [6], and to help track objects by using wireless concepts, optical tracking and ultrasound techniques [2]. Up to now, variety of navigation technologies have been developed for indoor environments such as sensors, Infrared (IR), Ultra Wide Band (UWB), Wireless Local Area Networks (WLANs), Wi-Fi, Bluetooth, Radio Frequency Identification (RFID), Assisted GPS (A-GPS) and so on [2,4,5]. Actually current navigation technologies cannot satisfy the challenging demands for most indoor navigation applications [6]. They all have limited capabilities (*i.e.*, low accuracy, unreliability, design complexity, low security and high configuration costs) to position, locate and track an object in an indoor environment (as will be discussed thoroughly in Section 2).

It is true that navigation systems in indoor environments will be highly useful to potential users. For instance, a person might need to attend a meeting in a building, but does not have much time, and would like to find the meeting room as fast as possible, without much effort. In today’s busy life, this kind of scenario is valid for most people, and it happens very frequently, as already discussed in our previous study [7]. It is true that people would appreciate a solution, which helps them save time and effort in such cases [7].

This paper presents an innovative, user friendly and low cost indoor navigation system called Near Field Communication (NFC) Internal, which takes advantage of NFC technology. The main idea of NFC Internal is to orient users having NFC mobiles and an installed indoor navigation application as well. While the application orients the user by receiving names or descriptions of the destination locations from the user; the mobile device collects the instant position of the user from the NFC tags those are spread all over the navigation area. Thus a user can determine his or her current position inside a building by touching the mobile device to the NFC tags.

**Figure 1:**
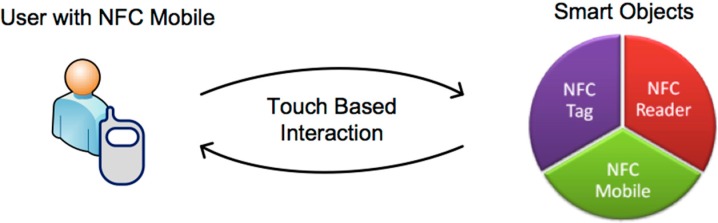
NFC interaction.

The triggering framework of the NFC Internal system is NFC technology that allows communication over short-range, mobile, and wireless conditions. NFC communication happens when two NFC capable devices are close to each other, essentially touching [8], as depicted in Figure 1. To do this, users use their NFC mobiles to interact with a smart object, which is either an NFC tag, another NFC mobile, or a NFC reader. After the touching activity, the NFC mobile may make further use of received data, or alternatively use available mobile services such as downloading a web page, initializing a web service connection *etc.* [8].

After a short introduction to the research topic, the remainder of this paper is organized as follows: In Section 2, a brief overview of existing indoor navigation systems and the essentials of NFC technology is provided. In Section 3, NFC Internal model issues are introduced first; system design and implementation of the model is given afterwards. We also present a case study and implement a prototype of an NFC Internal system in Section 4. Then in Section 5 we present a performance evaluation of the proposed model, and compare it with the existing indoor navigation techniques, and Section 6 concludes the paper.

## 2. Research Background

We will now point out some important concepts those are closely related with NFC Internal model. First, existing positioning technologies enabling outdoor and indoor navigation are briefly explained by using several comprehensive studies from the literature; the challenges of these systems are given as well. In the second subsection, NFC technology, its operating modes, and other NFC related issues are described for a clear understanding of the NFC communication aspects of the NFC Internal system. The brief research background will be helpful for understanding the significance of the contributions of NFC-based navigation systems compared to existing ones.

### 2.1. Indoor Positioning Technologies

In recent years, positioning systems have become a popular field in both academic and industry research and there already exist several research and commercial products in this area. Indoor navigation systems have become an especially hot research area recently, due to the unavailability of GPS in indoor environments, as explained in the Introduction section. With the aim of circumventing this lack of performance, an assortment of technologies has been tested and new designs generated for indoor navigation in the literature.

Positioning systems enable the appropriate device to determine its position, and make the location information available for position-based services [5]. Positioning system topology is an important issue for understanding and developing a positioning system. According to [9,10], four different system topologies are defined for wireless positioning systems: (a) remote positioning; (b) self-positioning; (c) indirect remote positioning and (d) indirect self-positioning. In case of a *remote positioning system*, there is a signal transmitter and several fixed measuring units. The signal transmitter is mobile, and fixed measuring units receive the transmitter’s signal. The location of the transmitter is computed in a master station after collection of all data from measuring units. In a *self-positioning system*, the measuring unit is mobile and it receives the signals of several transmitters placed in known places, and with the help of these signals, it can compute its location. In the case of *indirect remote positioning*, a self-positioning measuring unit sends the measurement results to the remote side via a wireless data link. A remote positioning unit can also send measurement results to a mobile unit, which is then named *indirect self-positioning*.

According to another study [3,11], indoor positioning techniques can be divided into two categories: *network dependent* systems and *device dependent (network independent)* systems. Network dependent navigation systems are based on networking technologies such as IR, sensors, ultrasound, WLANs, UWB, Bluetooth, RFID technologies, whereas independent navigation systems provide autonomous user positions such as A-GPS as an indoor GPS system.

**Figure 2:**
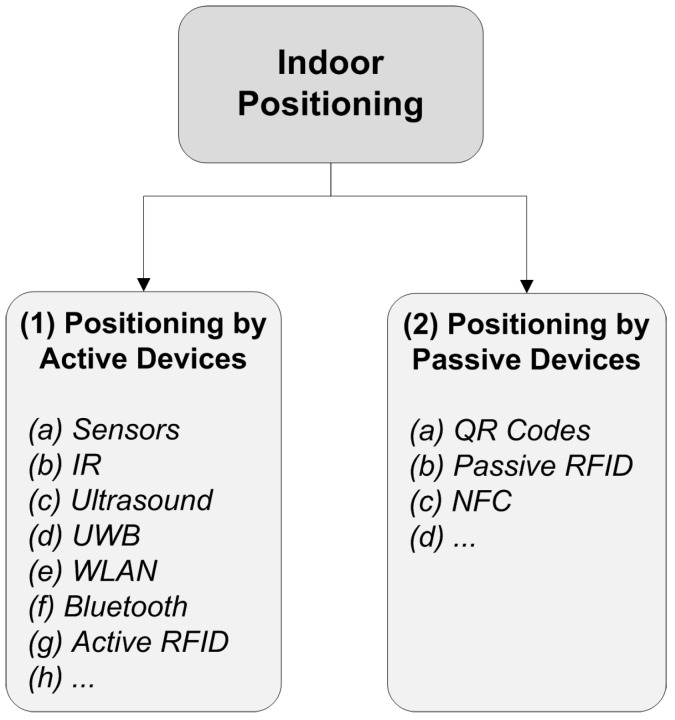
Classification of indoor positioning.

The study in [12] defines two approaches for indoor positioning: locating in relative coordinates and locating at choke points. Locating in relative coordinates requires usage of active devices (*i.e.*, devices that use their own power sources) for positioning, however passive devices can also be used for locating at choke points. Figure 2 illustrates a simple classification of indoor positioning technologies.

In case of *locating in relative coordinates*, reference transmitters determine the position of the user and the accuracy of positioning depends on the range and coverage of reference transmitters [12]. These systems usually calculate the position of the mobile object using the signal strength at reception time, which is expressed by the Received Signal Strength Indication (RSSI) technique. The technologies used in this method are IR, ultrasound technologies (e.g., Active Bat, Crickets, Dolphin [6]), UWB (e.g., Ubisense [5]), WLAN (e.g., RADAR, Ekahau, COMPAS [6,9]), Bluetooth (e.g., Topaz [5]), active RFID (e.g., LANDMARC [13,14]).

In case of *locating at choke points*, the sensors are located at fixed points, which provide location values in the network. In this approach, tagged objects including location coordinates determine the location [12]; some technologies for this approach are Quick Response (QR) codes, Passive RFID and NFC.

Infrared systems include several infrared transmitters located in various places of a building or closed environments, and these transmitters send their IDs, after which, a computing device with an infrared receiver uses these signals to determine its position. Active Badge developed by AT&T Cambridge was the first infrared positioning system [2].

More better and more accurate indoor positioning systems than Active Badge have been developed, which are called as ultrasound technologies, since infrared signals in a closed environment can easily be blocked by objects. In the case of ultrasound technologies, a large number of receivers and the sensitive placement of these receivers across the ceiling are strictly required [5], so in practice these systems have disadvantages in terms of scalability, ease of deployment and implementation costs [2].

UWB positioning systems use Radio Frequency (RF) signals for indoor positioning and provide high accuracy [2,5]. UWB positioning systems has their own limitations as well; an efficient indoor navigation system cannot be provided because of several technology problems such as low power emission, antenna mismatch, and possible external interference from other systems [11].

Another network dependent positioning system is Bluetooth, which is a simple short-range communication technology. These systems require expensive receivers and the position accuracy depends on the amount of receivers used. Thus Bluetooth-based indoor positioning systems may suffer in complex and changing indoor environments [5]. Similarly, Wi-Fi-based positioning systems require expensive access points to provide higher position accuracy.

Position accuracy depends on the type of the tags in the RFID case, which may be either passive or active; the positional density of these tags affects the accuracy as well. Passive tags are capable for small communication distances, whereas batteries are integrated into active tags to increase the communication distance. Available RFID-based indoor navigation models are mostly based on RFID tag usage and hence require vast amounts of tag usage for accurate positioning [15]. The major drawback of an active RFID-based indoor positioning system is the high cost of the active RFID tags, which does not provide a cost efficient solution [11].

Another popular existing technology for both outdoors and indoors is the A-GPS system, which is network independent. It expands the working area of the GPS technology to indoor environments by processing indoor GPS signals. However, the signal strength in indoor environments is sometimes too low [11].

Two types of positioning techniques are defined for positioning a person on a map [15]. A *fixed positioning method* determines the accurate position by using a sufficient number of assisting devices, such as satellites or antennas. In the contrary, the *dead reckoning (DR) method* calculates the estimated position by using the last preferred or estimated position, the direction, speed, and the time elapsed between the current time and the time of the last fixed position [15]. The major drawback of DR method-based navigation system is the poor performance caused by the computational errors. The positional calculation errors increase cumulatively when the calculation of the new position is based on the previous DR position.

**Figure 3:**
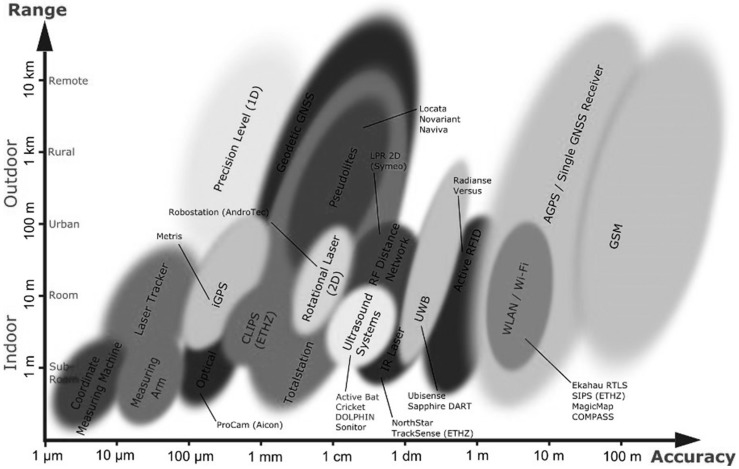
Current positioning systems according to their accuracy and coverage area (with permission from [6]).

Current technologies cannot meet the challenging demands of indoor positioning systems as stated in [6]. There is a great diversity in the accuracy and range of positioning systems for both outdoors and indoors; Figure 3 illustrates this diversity of existing technologies used for positioning systems. As seen in Figure 3, position accuracy varies according to the technology used for positioning.

Overall, a major limitation of indoor positioning systems is the higher cost and higher complexity of the system design, when compared with outdoor positioning systems. Additionally, most of the existing indoor positioning systems are far from providing an accurate position in large indoor environments [11,15]. Thus, there is a huge demand for a low cost, but efficient indoor navigation system, especially inside large structures consisting of many compartments, floors and halls.

NFC technology makes several contributions to the development of indoor positioning systems. However, no considerable and complete study on indoor positioning system using NFC technology exists yet. It is clear that more attention on NFC technology needs to be paid for the development of user-friendly, cost efficient and intelligent indoor positioning systems.

### 2.2. Near Field Communication

NFC is a bidirectional, short range, and wireless communication technology [16]; NFC communication occurs between two NFC devices only if they are within few centimeters of each other. For NFC communication, a 13.56 MHz signal that allows a bandwidth smaller than 424 Kbit/s is used. NFC technology operates in: (a) reader/writer; (b) peer-to-peer; and (c) card emulation operating modes, where communication occurs between a mobile phone on the one side, and a passive NFC tag, a mobile phone or an NFC reader, on the other side, respectively [8,17].

**Figure 4:**
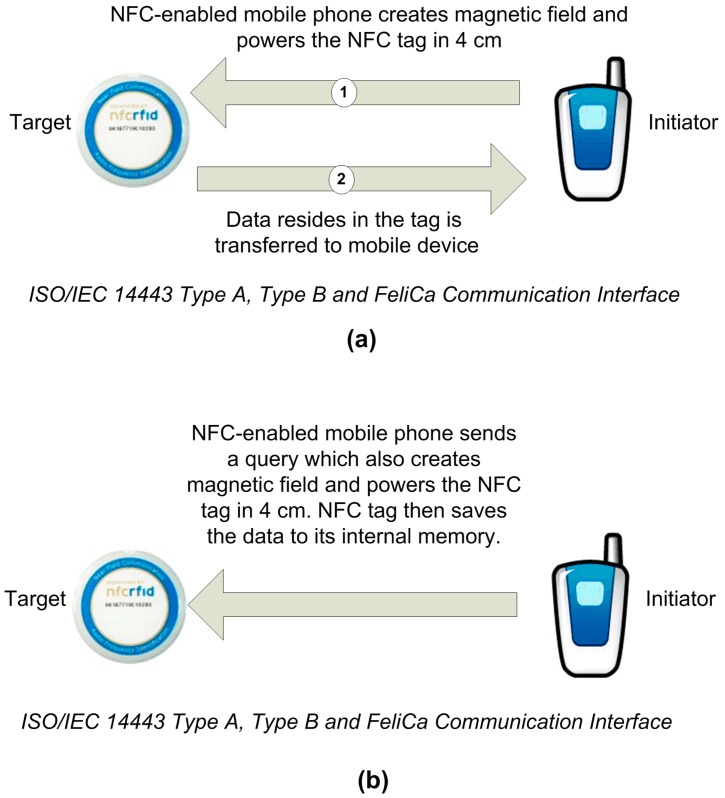
Reader/Writer Mode: (**a**) Reader Mode; (**b**) Writer Mode.

(a) In *reader/writer operating mode*, the NFC mobile initiates the wireless communication, and can read or modify data existing in NFC tags. This mode is compatible with the ISO/IEC 14443 and FeliCa standards for RF layer communication. When this operating mode is used, the NFC mobile is capable of reading NFC Forum [16] mandated tags, such as NFC smart poster tags. This process enables mobile users to receive the data stored in the NFC tag and take appropriate action thereafter (Figure 4). NFC Forum has defined four tag types based on their capacity and functionality, so that they are given designations as 1, 2, 3, and 4. NFC tag type formats are based on either ISO/IEC 14443 Type A or ISO/IEC 14,443 Type B or Sony FeliCa standards.

**Figure 5:**
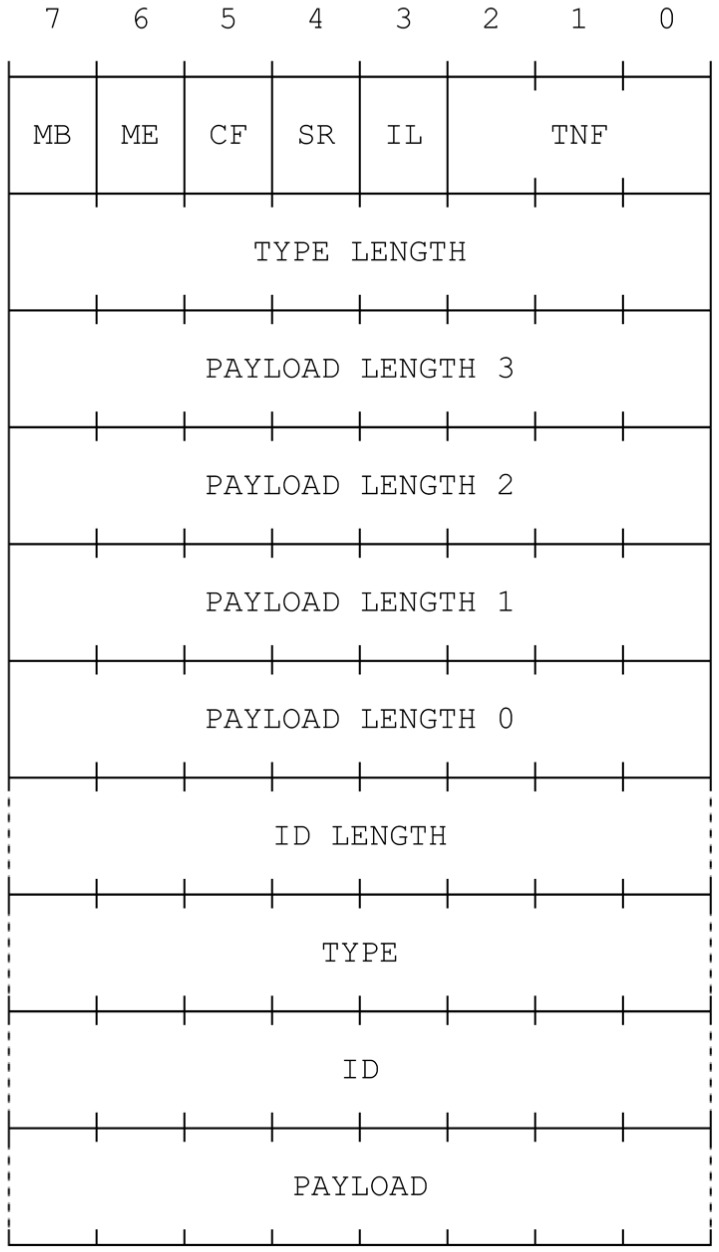
NDEF Record Structure (with permission from [18]).

To exchange information between a NFC mobile and NFC tags, the NFC Data Exchange Format (NDEF) is used, which is a data format specification standard defined by NFC Forum [16,17]. NDEF is a binary message that contains one or more NDEF records [18]. Each record consists of a payload up to 2^32^ − 1 octets in size as depicted in Figure 5. It is possible to chain records to support larger payloads. The most important field of the NDEF record is the Type Name Format (TNF), which is a 3-bits data. TNF describes the record type, and defines the structure and content of the record. NFC Forum defines various record types for specific cases; *smart poster*, *URI*, *digital signature*, and *text*.

**Figure 6:**
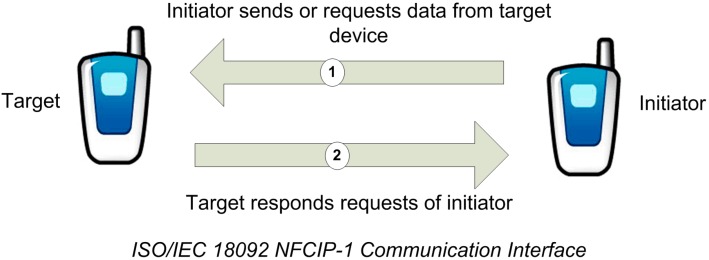
Peer-to-Peer Mode.

**Figure 7:**
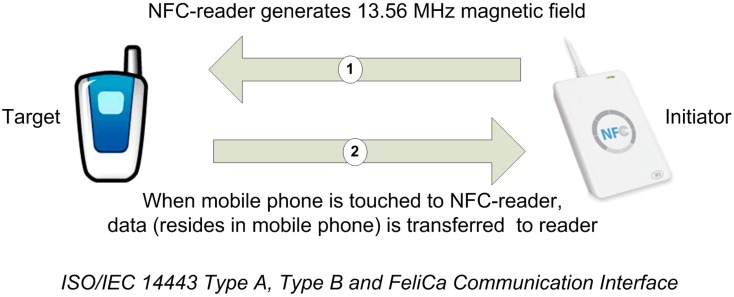
Card Emulation Mode.

(b) In *peer-to-peer operating mode*, two NFC-enabled mobile phones can establish a bidirectional connection to exchange information among them (Figure 6). They can exchange data that resides on the mobile phones such as virtual business cards, digital photos, or other.

(c) In *card emulation operating mode*, a NFC-enabled mobile phone acts as a smart card and stores private values such as credit card, debit card, identity, passport information, etc. called a secure element (SE) on their secure areas. As the user touches the mobile phone to an NFC reader, the NFC reader initiates the communication and gets the required valuable information from then NFC mobile (Figure 7).

As described and illustrated, *touching* is the essential part of the interaction for all NFC technology operating modes. Touching occurs when the NFC mobile is brought into NFC communication distance with another NFC-enabled object. The required range is very small, being between 0 and a few centimeters [17,18]. Touching is a natural action for human beings, and accordingly maybe the mostly used interaction technique, since all that a user must do is physically touch the NFC mobile to another NFC-enabled object. Additionally, the communication after touching is inherently secure since the NFC communication is performed over a short distance.

**Figure 8:**
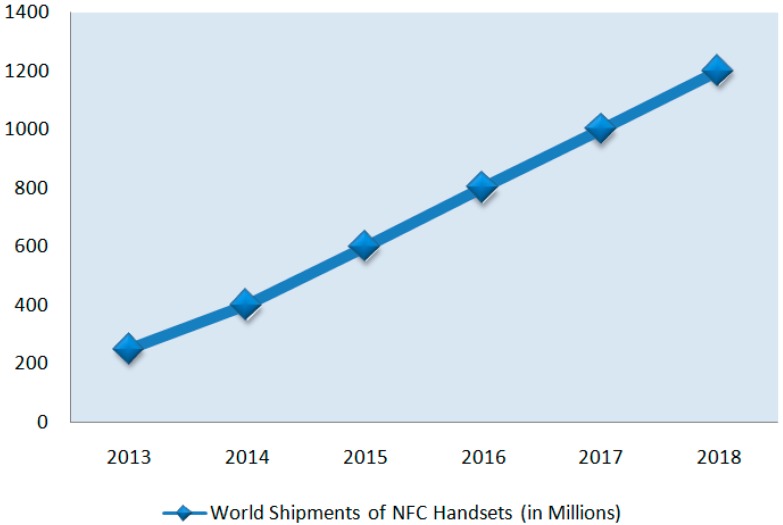
Forecast for World Shipments of NFC Handsets, in Millions (adapted from [19]).

Up to now, many NFC trials in diverse application areas have been conducted over the world. All existing trials reveal the fact that after the NFC application usage NFC mobile can become safer and speedier [17]. According to a forecast from IHS Technology (*i.e.*, one of the world’s major consulting sources for research and analysis in technology, media, and telecommunications industries), NFC will be included in 64% of the mobile phones shipped in 2018, up from 18.2% in 2013 as seen in Figure 8 [19].

Researchers add that global shipments of NFC mobiles in 2018 will be four times higher than in 2013. With the increasing processing power of mobile phones and Internet access capability; more innovative services will be enabled by NFC technology. Another analysis states that the majority of smartphone manufacturers have started to adopt NFC and hence payment technology in their products as a “*de facto* standard” [19].

NFC technology brings simplicity to communication, enables easy data delivery, and provides information sharing. Additionally, it offers new opportunities for service providers such as mobile network operators, financial institutions, transport companies and merchants to enable faster transactions, the use of digital money, and additional services [20,21,22,23].

## 3. NFC Internal

This study aims to create an innovative, user-friendly, simple, and cost effective NFC-based indoor navigation system called NFC Internal in order to eliminate the current problems of indoor navigation. Firstly the generic usage model of NFC Internal is described, and then the system components of the NFC Internal system (*i.e.*, Map Tag, Location Tags, and NFC Internal Application) are explained in detail.

### 3.1. Generic Model of NFC Internal System

**Figure 9:**
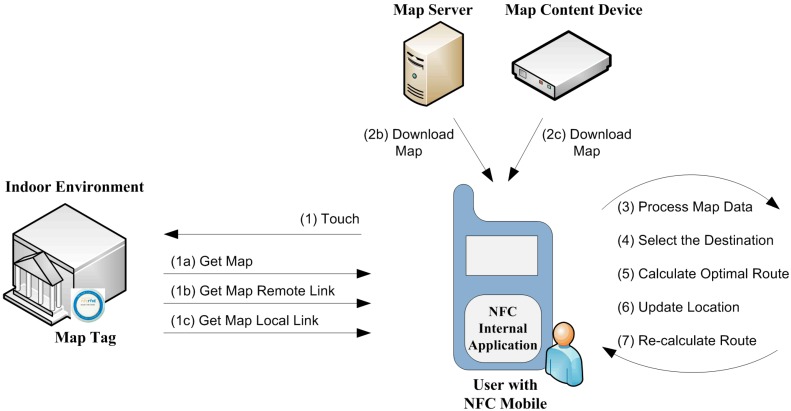
Generic model of NFC Internal.

Figure 9 illustrates the generic model of the NFC Internal system. In order to activate the system, the user needs to NFC-touch the NFC-enabled mobile device to the Map Tag, which contains either: (a) an encoded map data of indoor environment; (b) a link to download a map from a remote device called Map Server (a server storing the map data); or (c) a link to download a map from a local Bluetooth-enabled device called Map Content Device. At least one Map Tag needs to be placed just before entering the indoor environment (e.g., campus, school, hospital, shopping center and so on).

After touching the tag, the NFC Internal application runs automatically, retrieves the data from the tag and processes the map afterwards. If the user did not previously install the NFC Internal application, the mobile device is directed to the application installation automatically after touching the Map Tag. As the user specifies the name or description of the destination, the NFC Internal application calculates the optimal route to the destination. When the user navigates through the halls, he/she can touch the Location Tags, which contain the location data of the tag coordinates. These Location Tags are spread all over the building and update the user’s current position on the map. Then the user gets instructions from NFC Internal application to reorient the route to the destination, or to create a new optimal route, as required.

As an extension, NFC Internal is coupled with an outdoor navigation system to provide higher functionality. After the user executes NFC Internal and provides the destination address, NFC Internal implicitly makes use of GPS-based navigation, and navigates to the entrance of the destination indoor environment (*i.e.*, Map Tag). If the user already downloaded a map of the indoor environment using NFC Internal, he/she can select a destination from the indoor environment and navigate to it using NFC Internal even he/she is in an outdoor environment.

**Figure 10:**
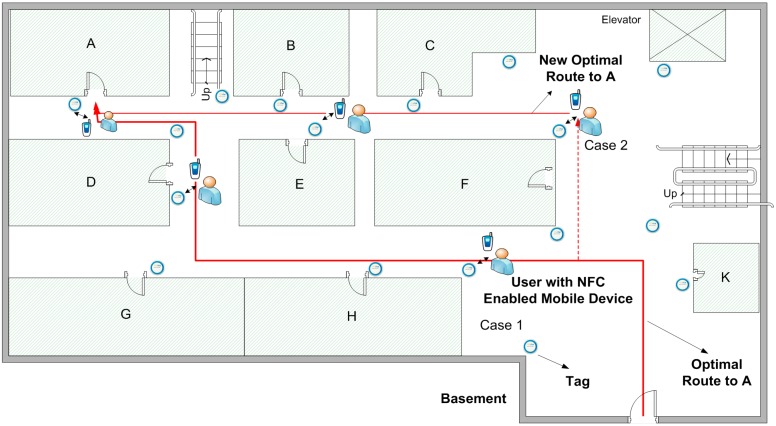
Navigating to a destination.

An example might be beneficial. Consider a student currently living at home who needs to meet with an advisor to perform a course registration process. In order to navigate from home to the advisor’s office, the student needs both a GPS-based navigation application and the NFC Internal application. However, if the student already downloaded the indoor map to the NFC Internal device, he/she can navigate to the advisor’s office with only the NFC Internal application. When the student inputs the advisor’s office as a destination point in NFC Internal, the application orients the student through the university campus by GPS. As the student reaches the university campus, NFC Internal starts indoor navigation. As shown in Figure 10, let’s imagine that the student wants to be directed to Office A, and navigates inside the building.

He/she touches the first Location Tag seen, after which the location data on the tag is transferred to the NFC Internal application (Case 1). The application thus finds out that the student is on the intended route and updates the user location on the screen. When the user reaches the destination point, navigation ends. However, if the student diverges from the route and touches a Location Tag (Case 2), then NFC Internal calculates a new optimal route to the destination.

### 3.2. System Components of NFC Internal System

As briefly mentioned above, the components used in NFC Internal System are the following: (a) Map Tag; (b) Location Tags; and (c) NFC Internal Application.

(a) Map Tag: There are three options for accessing map data using Map Tags, as seen in Figure 9:
  1. The map data is encoded completely into the Map Tag (Figure 9, steps 1 and 1a),
  2. The Map Tag includes a link to the remote device—called Map Server—as a web server containing the map data. After getting the related link from Map Tag, the map data can be downloaded to the user’s mobile device (Figure 9, steps 1, 1b and 2b), or
  3. Map Tag includes a link to the local device called a local Map Content Device (*i.e.*, a local device containing the map data). After getting the related link from Map Tag, the map data can be downloaded to the user’s mobile device (Figure 9, steps 1, 1c and 2c).
(b) Location Tags: In order to navigate through indoor environments, NFC Internal needs map data to calculate all accessible paths. In NFC Internal system, a vector spatial data structure, which uses 2D Cartesian (*x*, *y*) coordinate pairs is preferred for the location information on a Location Tag. Usage of vector spatial data allows efficient encoding, and network linkages can be efficiently employed [24], hence, it is more useful for accurate positioning. In the NFC Internal system, each indoor environment may require different numbers of Location Tags. These tags are placed on many places such as rooms, elevators, and stair entrances as well as throughout corridors. The number of tags used depends on the size, structure, and complexity of the structure. As explained in the Performance Evaluation section, expectations of higher accuracy also require a higher density of Location Tags. Each Location Tag includes location data in NDEF message format. This location data is comprised of building identifier data, floor identifier data and vector spatial data (*x*, *y*).
(c) NFC Internal Application: the NFC Internal application is the smartphone application developed specifically to implement the NFC Internal navigation system. The application needs to be installed on a smartphone and has following functionalities:
  1. Get the destination data from the user,
  2. Calculate the shortest path between the current position and the destination taking the user's navigation preferences into account,
  3. Provide an optimal route to the user by giving real-time instructions,
  4. Update the position of the user when the Smartphone is touched to a Location Tag,
  5. Re-calculate the shortest path between the current position and destination after the Smartphone is touched to a new tag,
  6. Calculate the user’s approximate position using DR technology by calculating the user’s steps and estimating the walking direction, and
  7. Make use of GPS based navigation application for outdoors, if required.

NFC Internal pays attention to user preferences. On travelling to a different floor, a user may prefer to use elevators, escalators or stairs, for example. Depending on the user’s preference, the NFC Internal application calculates the optimal route to the destination point using a shortest path algorithm.

**Figure 11:**
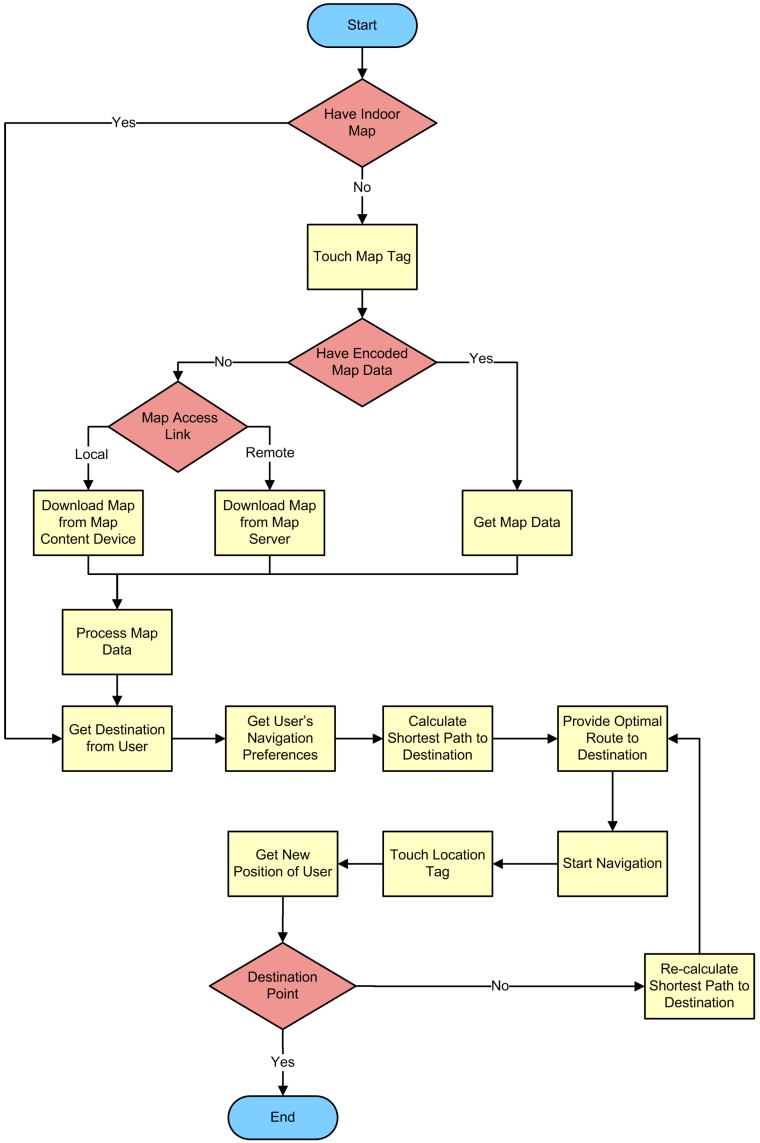
Flowchart of the NFC Internal application.

As seen from the flowchart of NFC Internal application (Figure 11), the NFC Internal application is started after the user touches a Map Tag or Location Tag. If the application is not already installed on the Smartphone, the user either should either install it manually or touch a Map Tag to install it automatically. If the map is not already downloaded to the NFC Internal application, the user touches the mobile to the Map Tag; after which the mobile device gets the map as depicted in Figure 9.

After the download process, the NFC Internal application asks the user to enter the destination. Several alternatives exist for entering the destination. The user may either use a search tool provided by the application, or select from a given list. Then the application quickly computes the best route by using the algorithm. After the route is calculated, the application starts to orient the user towards the destination. Then, the calculated route is displayed on the screen. Based on the specific implementation of NFC Internal, verbal instructions may also be provided.

As the user navigates along the path, he/she can touch any Location Tag on the way to validate or update the position. Position information of the Location Tag, which obviously shows the position of the user, is sent to the application. The NFC Internal application makes use of this knowledge by checking it against the intended route.

## 4. Case Study and Prototype Implementation

Shopping malls are typically large and most have complex inner structures. The building architecture and the placement of stairs are designed so that the customers are obliged to travel—and browse—as much distance as possible; it is assumed that the customers may potentially make purchases even if they do not visit the mall for purchasing those specific items. On the contrary, the visitors that may not have much time and do not wish to spend too much time there expect to be able to visit a specific shop within the mall. NFC Internal is definitely a time, effort, and money saver in such cases. This is especially true for first time visitors. Currently, some shopping malls provide computer-based guidance services such as interactive kiosk machines located in certain places, however reaching/finding that kiosk machines is usually a problem in itself in complex shopping centers. Human-based assistance services are one-time only, and as a matter of fact do not help too much and do not provide on-line help after some distance is travelled in the mall.

**Figure 12:**
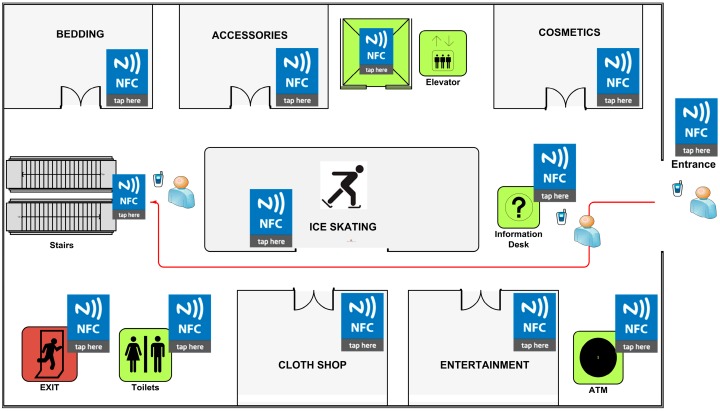
Shopping Mall Map—Floor 1.

**Figure 13:**
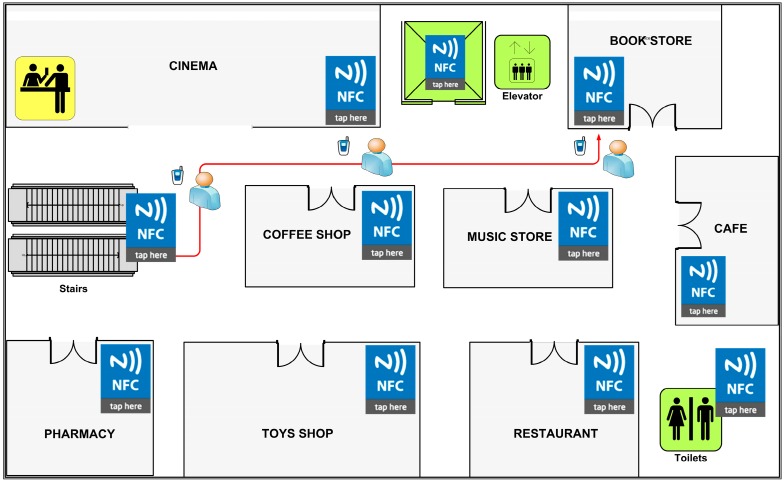
Shopping Mall Map—Floor 2.

We developed an NFC Internal prototype for testing purposes. In this section, we provide an explanation of an NFC Internal system prototype, which is based on a real life shopping mall case. The maps of the shopping mall are illustrated in Figure 12 and Figure 13.

Map Tag is located at the entrance of the shopping mall as established by the NFC Internal system requirements (Figure 12) and map data is encoded into the Map Tag. Location Tags are placed near shop doors, walls, and corners of the building structure. The amount of Location Tags spread on the walls was chosen to be high to increase the accuracy; and many redundant tags are placed on central points to ensure high visitor satisfaction by avoiding any possible queuing.

According to the scenario, a visitor enters the shopping mall for the first time and aims to purchase a present for her very best friend from a Bookstore which is on the second floor of the complex, as seen in Figure 13.

**Figure 14:**
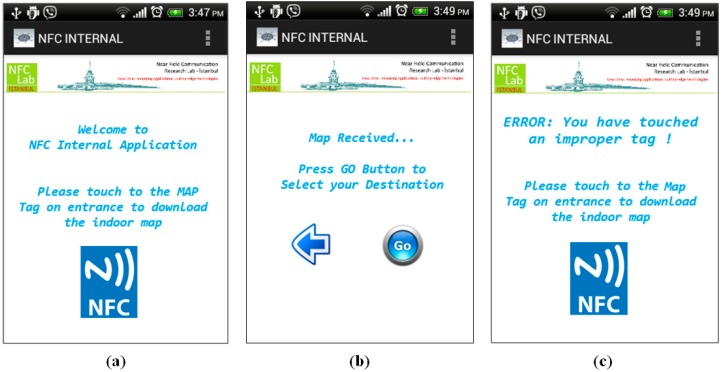
Initiating NFC Internal—Shopping Mall.

*Step 1*: The user touches his/her mobile to the Map Tag located at the entrance of the shopping mall. The application is not installed on the user’s Smartphone so it is directed to the application page of the application. After the application is installed, the map is loaded onto the Smartphone immediately as seen in Figure 14a,b.

Actually user needs to touch the right/proper tag, which carries the map information of the complex. If the user touches another NFC tag for some reason, the NFC Internal application displays an alert message to the user as seen in Figure 14c.

**Figure 15:**
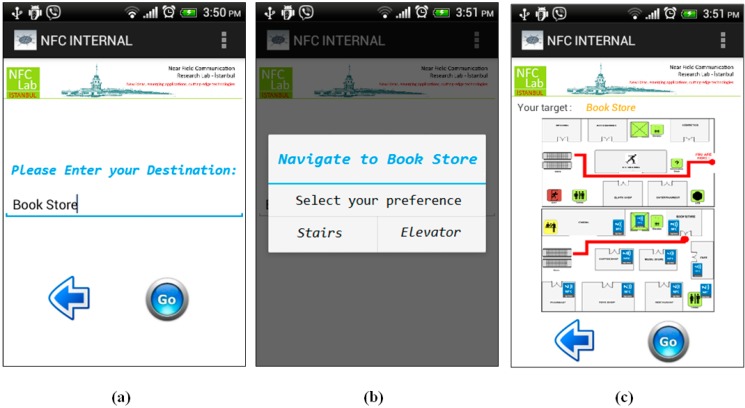
Navigate to Book Store—Shopping Mall.

*Step 2*: User enters the name of the store where she wants to go; in our case the destination point is Book Store as seen in Figure 15a.

*Step 3*: User preference is required at this point. Before calculation of the optimal route, the NFC Internal application asks for the user preference since it is not saved it in the application preferences as shown in Figure 15b. Then the application calculates the optimal route to the Bookstore, which is located on the second floor, and gives the appropriate instructions to the user as seen in Figure 15c.

**Figure 16:**
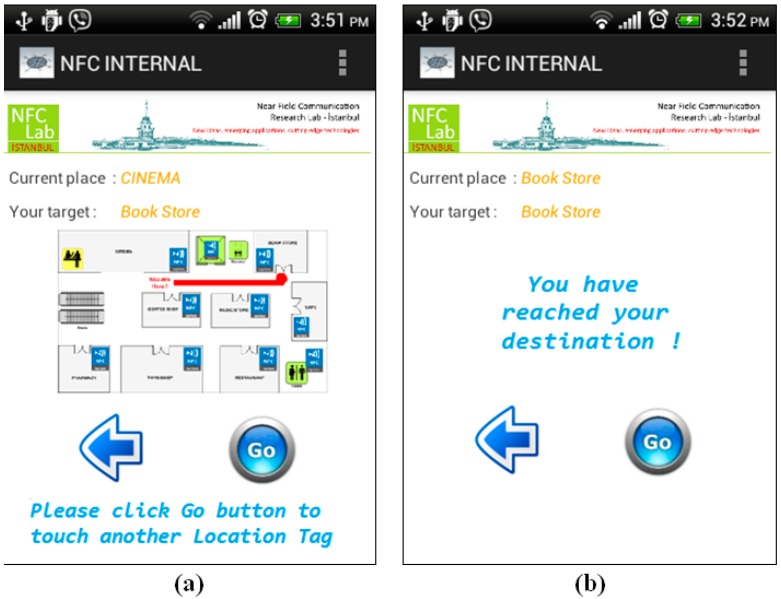
Instructions given by Location Tags at the Cinema and Bookstore—Shopping Mall.

*Step 4*: It should be remembered that during navigation, the NFC Internal application orients the user to the destination point by giving instructions, as Location Tags spread all over the building are touched. After getting the first instruction, the user starts to navigate within the shopping center and goes up to the second floor using the stairs. After getting to the second floor, the user touches another Location Tag near the Cinema that is on the previously calculated route. The application updates the position of the user on the map, and notices that user is still on the correct route, so it gives updated instructions to the user as seen in Figure 16a.

*Step 5*: As long as the user navigates on the intended route, the NFC Internal application uses the calculated shortest path information. On the contrary, a correction is required if the user diverges from the given route. The user also confirms that he/she has reached the destination point by touching the Location Tag placed at the Bookstore as seen in Figure 16b.

## 5. Performance Evaluation

This section consists of a performance evaluation of NFC Internal and a comparison with other existing indoor navigation systems (e.g., IR, ultrasound systems, UWB, WLAN, Bluetooth, active RFID *etc.*). We evaluate the performance of NFC Internal based on the criteria defined in [5] which presents considerable system performance and deployment criteria to evaluate and compare indoor navigation systems from the user’s point of view. All these criteria are fully focused on user preferences and experiences, which will provide a beneficial road map to evaluate the NFC Internal system as well. According to [5], the evaluation is to be performed according to the following criteria:

(1) *Security and privacy*: Privacy has always been an important concern for the users. Privacy in navigation systems involves keeping the user’s position private. When a user navigates to a destination, the user’s location is retrieved from the passive NFC tags by the Smartphone and processed by the NFC Internal application afterwards. Unless the Smartphone gives its location information to an outsider, no one can access this private and sensitive information. Moreover, as the user touches an NFC tag on the way, the communication is one way and to the Smartphone only; hence no data is passed to the tag. This implies that the tag does not have any information—or even and idea—about the identity of the user. As a result, it is ensured that the privacy is preserved in the NFC Internal system.

*Comparison with other technologies:* Existing indoor solutions mostly use RF-based technologies; ultrasonic technologies are preferred sometimes. The location of the navigating object is determined by calculating the distance of the object using the signals coming from transmitters. In signal-based systems invasion of privacy, theft, accessing sensitive information from transmitters, and corruption of this sensitive information are all challenging issues, when compared with NFC technology.

(2) *Cost*: In order to enable the NFC Internal system, the following components needs to be prepared: Map Server or Map Content Device if the MAP will be installed to either one, NFC tags as Map Tag and Location Tags, and Smartphones. We analyze cost of each item in detail as follows:

- Map Server or Map Content Device: Please remember that the map data might be encoded to the Map Tag. On the contrary, if the map will be installed to a remote server or local device, a computer with average performance shall be prepared accordingly, in which the server will have a reasonable cost.
- NFC Tags: One or some redundant number of Map Tags will be installed at the entrance, and a vast amount of Location Tags will be installed all over the building or campus. The cost of tags can be analyzed based on:
  ○ Purchase cost: As of today, one NFC tag costs less than 1$ if purchased in bulk. Therefore, using a few hundred, or even a thousand tags represents a negligible cost.
  ○ Installation cost: Map Tags as well as Location Tags should be encoded and then placed at the design locations. Encoding tags is not a time consuming process; installing all the tags of one NFC Internal project requires only one or two days, even for a large area.
  ○ Maintenance cost: Fixing a damaged tag implies replacing it, since it is much simpler than dealing with troubleshooting the problem. NFC tags are passive components that do not contain any batteries hence these do not require replacement with respect to power failure. Since NFC tags do not wear out as time passes, they therefore can be used nearly forever, unless they are damaged or destroyed by environment—or human originated—conditions. In order to check whether they operate smoothly or not, they shall be periodically inspected, which requires some minor effort. Simply reading the content of each tag on any NFC mobile will perform this process; if the mobile reads the content of the Location Tag, it is performing correctly; otherwise the tag shall be immediately replaced. Please note that any Location Tag with an error will cause only a local problem, and will not affect the overall system. We can conclude that maintenance of the NFC Internal system has a trivial cost.
- Smartphones: Users those intend to use NFC Internal will already own a mobile phone, hence resulting in no additional cost.

*Comparison with other technologies:* Each alternative model to NFC Internal includes a device ownership as well as maintenance cost on the user side. In terms of purchase and installation costs, comparison of existing solutions has already been performed in previous studies [2,5,9]. These costs generally vary between moderate and high for all existing solutions. As seen from the studies, the hardware and installation costs of the systems increase when higher performance is expected from an indoor navigation system; such systems need extra infrastructure and configuration issues to provide higher performance. For example, sensor-based navigation systems require extra infrastructure, which can be expensive and complex; fixing sensors in different places and their proper placement is an important issue. Also, GPS-based indoor navigation systems are expensive and complex solutions since they also need large infrastructure to support the high performance and accuracy for location measurement. Similarly the operating cost of high performance UWB-based indoor navigation systems is high. Also, existing solutions mostly need professional engineers/experts to support the operation of the system and provide maintenance for the system, which means additional costs.

(3) *Performance*: With respect to the performance evaluation, accuracy and precision are the two main parameters for indoor navigation systems [5]. Accuracy is defined as the distance between the calculated and the actual values, whereas precision is defined as the achievement of position estimations with respect to some predefined accuracy. The accuracy of the NFC Internal system depends highly on the number and proper placement of the Location Tags. As an example, consider two neighbor Location Tags, T1 and T2. The user first touches T1 and the application locates his/her position on the map automatically; afterwards he/she moves away from that location and walks towards T2. Until the user touches T2, the application predicts the user’s approximate position using DR technology by calculating the user’s steps and estimating the walking direction, hence the system works with a possible distance error of x meters and consequently, the accuracy is x meters. In order to decrease the distance error, and hence improve the accuracy in the NFC Internal system, decreasing the distance between the neighboring tags to some lesser value shall be considered. There is a limit to the size of this distance though, as users do not want to be touching neighbor tags repeatedly at very short distances. Hence, an optimum distance shall be calculated. As another issue, critical locations—such as elevators, stairs—need to be tagged as well. On the other hand, the precision of location awareness in the NFC Internal system is almost 100%, because when a user touches a tag, the exact location is retrieved immediately.

*Comparison with other technologies:* As discussed in [5], none of the existing technologies—other than NFC Internal—can satisfy the system requirements of performance and accuracy. Also, there is a trade-off between coverage area and accuracy for indoor navigation systems. For example, WLAN technology can provide navigation services with high accuracy for large indoor environments, however UWB can only provide highly accurate position estimations for small areas. In the case of active RFID-based indoor navigation systems, the signal strength sometimes drops between the tag and the antenna, hence the accuracy level depends on the proper placement of readers in the indoor environment. In short, accuracy levels and error distances vary for most navigation technologies as already presented in [2,6], and in the research background section of this study. We have mentioned this diversity using Figure 3 as well. None of the existing solutions provide 100% location precision awareness when compared with NFC Internal.

*(4) Robustness and fault tolerance*: NFC Internal system is so robust because the reading lifetime of a passive NFC tag is nearly unlimited, and the probability that it will malfunction during its lifetime is negligible as well. Additionally, even if a fault occurs in a tag, the overall system maintains its performance because the fault in one tag does not affect the remaining system in any way. Only the users who cannot touch that tag will suffer temporarily from a smaller accuracy. We can easily conclude that the system is tolerant to faults.

*Comparison with other technologies:* The robustness of the indoor navigation systems is affected or influenced by some elements (*i.e.*, metal, florescent lights, sunlight, noise), reflection of signals from obstacles, limited battery power and so on, which also decrease the accuracy and performance level of the systems. Sensor-based navigation technologies have limited battery power, and provide less accuracy as the system runs out of battery power. Obstacles in the indoor environment decrease the accuracy of the system, which is an important concern, especially for IR and ultrasound systems since line-of-sight is highly required in these systems. Signal strength in WLAN-based indoor navigation technologies are also highly affected by various elements such as overlapping of access points, movement and orientation of the human body, walls, doors and so on. In the case of NFC technology, the presumably unlimited lifetime of the tags and low probability of a fault in a tag increases the robustness of the system.

(5) *Complexity*: Aspects of the complexity for indoor navigation systems given in study [5] are the human intervention/efforts during the deployment and maintenance of the indoor navigation system, and the required computing and processing time of the device carried by the user to determine his/her position. Because of the effort required for installing all the system components such as the Location Tags and the Map Tags, human intervention during the initial deployment of the NFC Internal system is very high; but on the contrary, the human effort required for maintenance is very low after the deployment process. With respect to the users; they won’t waste time using the system, since the required processing time for any action such as positioning, calculating routes and so on is very low, and can be measured in milliseconds. At this point, it should be noted that if the Location Tags are spread more densely, users may waste their time touching the tags too frequently, possibly to increase their position accuracy. In conclusion, we can say that the complexity of NFC Internal is average.

*Comparison with other technologies:* Complexity evaluations have also been performed in previous studies [5,9]. Existing technologies mostly have moderate or low complexity levels like the NFC Internal case [5].In terms of effort for installing system components, almost all indoor navigation technologies require better indoor planning since the system components (e.g., sensors, receivers, access points, tags and readers) need to be placed properly. Especially for sensors, IR, ultrasound, WLAN, and active RFID technologies, as the coverage area increases, the complexity becomes an important concern. In terms of the second aspect, lower computing time and higher processing capability are always desirable for any navigation solution; the limited battery power of positioned devices and their processing capabilities need to be addressed.

(6) *User preference*: As given in [5], since user comfort is a significant issue, indoor navigation systems should also consider the users’ requirements of infrastructure, devices and software; user friendly and easily learnt systems are preferable. In NFC Internal, users need to carry only their Smartphones, but nothing else. Any user who aims to use NFC Internal is required to install the NFC Internal application to their mobile phone. When a user who did not install it prior to touching the Map Tag, the device is directed to the appropriate application store—which is specific to the Mobile Operating System of the Smartphone—automatically.

*Comparison with other technologies:* In almost all indoor navigation systems, users need to obtain the hardware and software for using the system; moreover they need to carry/own the corresponding positioning devices. The system components needs be small, wireless, lightweight and low power consumption (or alternatively have long battery life) to offer responsive and accurate navigation services.

(7) *Commercial availability*: The design details of the NFC Internal are being made publicly available in this research paper, which should be much valuable for future improvement of custom NFC-based indoor navigation systems.

*Comparison with other technologies:* Today there are several alternatives for indoor navigation, which are commercially available as well. Active Badges, Active Bats, Crickets, RADAR, COMPASS are not commercially available [5].

(8) *Limitations*: NFC Internal does have some limitations. First of all, a user who aims to use the system must have an NFC-equipped mobile. We do not assume this as a discouraging requirement, since the acceptance rate of NFC in Smartphones is very high, and it is constantly increasing. A solution to overcome this limitation may be lending NFC-enabled, but possibly low price and capability Smartphones or other suitable NFC-enabled mobile devices to users while they are at a site.

Real time positioning cannot be provided in NFC Internal system. The DR method is used by NFC Internal to calculate an approximate location when the user is moving between Location Tags, and fixed positioning will be performed immediately after the user touches to the next tag.

If the density of placed tags is low with respect to the user traffic, queues may occur in front of many tags. Additionally, queues will possibly occur in front of Location Tags located a certain key places such as elevators. To prevent queues in such places, redundant tags can be deployed so that a visitor can use another spare tag if another user is already using the other tag.

*Comparison with other technologies:* As already mentioned, existing indoor navigation solutions have diverse limitations in terms of security, cost, performance, robustness, complexity, user preference and commercial availability. In particular there is a great trade-off between accuracy and cost between existing indoor navigation solutions, as systems with high performance also have higher cost [5]. Hence a desirable and reliable solution will be highly appreciated.

(9) *NFC Internal prototype test results*: As presented in Section 4, we provide and describe an NFC Internal system prototype for testing purposes, which is based on a real life shopping mall case. During the implementation of the NFC Internal system prototype, we measured the prototype system’s performance in light of the performance parameters mentioned above, and achieved some valuable results as seen in Table 1.

**Table 1:** NFC Internal prototype test results.

Parameter	Results
Security and Privacy	The user’s location was retrieved from the NFC tags and processed by the NFC Internal application on the Smartphone afterwards. During tests, users indicated that they feel secure while touching the NFC tags and the privacy of their sensitive information is highly ensured.
Cost	The prototype had only the initial deployment cost. Since location information is encoded to one NFC tag, so no Map Server cost exists. Purchase cost of NFC tags plus additional materials such as shields was approximately $50. During installation of NFC Internal, one person was employed and the cost of the initial deployment was approximately $50.
Accuracy	Location Tags were spread frequently in halls of the shopping mall and properly aligned; the distance between two NFC tags was 10 meters. However, during the tests, it is seen that users did not touch every Location Tag. Instead, after touching a tag, they walked without touching the next few location tags, and then touched a next tag. Thus, using NFC technology gave around 15–20 m of location accuracy. However, it is seen that when users were not sure of their location, they immediately touched the closest tag.
Precision of Location Awareness	The exact location of the places are encoded to NFC tags. Thus the exact location was retrieved by NFC Internal after touching a tag. Hence, the precision of location awareness in our NFC Internal prototype is 100%.
Robustness and Fault Tolerance	A few tags were intentionally damaged to test the robustness of the system. A few users touched the damaged tag and realized that they cannot get their location. It was seen that, they then touched the closest tag after the damaged tag, and then continued their navigation without any problem. Thus the system’s tolerance to faults was 100%.
Complexity	Human intervention/efforts during the installation phase of the system was very low, because the indoor environment was small-medium scale. However, it is anticipated that, in larger indoor environments, the complexity will grow exponentially. Processing time for determining the position depends on the time required for getting data from the NFC tag and processing it on the Smartphone. The reading speed from NFC tags varies from tag to tag. In the prototype, it is seen that reading speed from the NFC tag and the time needed to process the data on a Smartphone were very short, approximately less than 0.5 s.

## 6. Conclusions

Global Positioning Systems, commonly used for navigation, are very useful in outdoor environments, and hence are normally known as outdoor navigation systems. GPS satellite signals are obstructed by the structures in closed environments, resulting in GPS positioning being impossible in indoor environments. Several indoor navigation options have already been defined to overcome the GPS limitations in indoor environments, but they suffer from security, cost, and performance problems, *etc.*

In this study, we present an innovative, reliable, and ubiquitous indoor navigation solution called NFC Internal, an NFC-based indoor navigation solution to overcome the aforementioned problems. NFC Internal orients users carrying Smartphones. It makes use of NFC technology that enables seamless data transfer just by touching a Smartphone to tags spread over the indoor area. Hence, users can get their position fixed during their tour of a building or a complex such as a university or medical campus. NFC Internal is composed of components as Map Tag, Location Tags, and an NFC Internal application installed on the Smartphone. Our study includes the system requirements and design details, a prototype development to study the viability of the NFC Internal model in a shopping mall case study and concludes with a thorough performance evaluation.

NFC Internal is evaluated in terms of security, privacy, cost, performance, robustness, complexity, user preference and commercial availability and compared with existing technologies accordingly. By making use of NFC technology, the following significant advantages over existing indoor navigation systems are provided:

- NFC Internal provides secure and private navigation due to the nature of NFC technology (no personal data is leaked out of the Smartphone),
- NFC Internal is cost effective (the purchase, configuration and maintenance costs of each system component are negligible as explained in Section 5, Part 2 Cost and Part 9 NFC Internal Prototype Test Results),
- NFC Internal provides accurate positioning of users (the accuracy of the system depends completely on the number and proper placement of NFC Tags which can be handled easily during the configuration as explained in Section 5, Part 3 Performance and Part 9 NFC Internal Prototype Test Results),
- NFC Internal provides high robustness (the lifetime of NFC tags is essentially unlimited and occurrence of a fault on a tag does not affect the rest of the system, which continues to work with the remaining tags),
- NFC Internal supports high location awareness precision (*i.e.*, when a user touches a tag, the exact location is retrieved immediately.

Overall, NFC Internal is shown to be a simple, non-expensive, seamless, secure, and ubiquitous indoor navigation system and to have significant advantages over its alternatives.
